# Supplementary material for: Coherent microwave generation by spintronic feedback oscillator
Source: Sci Rep. 2016 Aug 1;6:30747. doi: 10.1038/srep30747 (PMC4967853; doi:10.1038/srep30747)
Supplement: Supplementary Information [file srep30747-s1.pdf]

Supplementary Information

Coherent microwave generation by spintronic feedback oscillator

Dinesh Kumar[1], K. Konishi[2], Nikhil Kumar[3], S. Miwa[2], A. Fukushima[4], K. Yakushiji[4], S. Yuasa[4], H. Kubota[4], C. V. Tomy[1], A. Prabhakar[3], Y. Suzuki[2], A. Tulapurkar[5]

Supplementary Note 1: Micromagnetic simulations:

The micromagnetic simulations were carried out by using MuMax3 program. The parameters used in the simulation are given below:

Saturation Magnetization	800 emu/cc
Damping	0.01
Exchange constant	13 pJ/m
Shape	Elliptical (500 nm x 300 nm x 3nm)
Cell size	5nm x 5nm x 3nm
Time step	0.1 ps
Anisotropy	Hx=15.6 Oe, Hy=47.5 Oe
Temperature	300 K

The values of anisotropy constants were adjusted so as to get a close match of the FMR frequencies obtained from simulation with the experimental data (Fig. 1b in the manuscript). The feedback was implemented in the following way:

The resistance of the MTJ depends on the magnetization direction of the free layer as:

$$R = R_p + \frac{\Delta R}{2}(1 - m_x) \qquad (1)$$

where *Rp* is the resistance of the parallel state and ΔR is the difference between the resistances in anti-parallel and parallel states. If the magnetization direction of the free layer is oscillating, dc current passing through it produces ac voltage given by:

$$V_{ac}(t) = I_{dc}\,\Delta R_1(t) \qquad (2)$$

where ΔR_1 is the oscillating resistance given by $-0.5\Delta R m_x(t)$. This oscillating voltage produces an oscillating current in the co-planar wave guide given by $V_{ac}/(R_T+R_{MTJ})$, where R_T is the resistance in which the feedback circuit is terminated. (We assume R_T is same as the characteristic impedance of CPW=50). R_{MTJ} is the average resistance of MTJ. Thus the oscillating current flowing through the CPW is given by:

$$I_{ac} = -\frac{I_{dc}\,\Delta R\, m_x}{2(R_T + R_{MTJ})} \qquad (3)$$

The magnetic field created by the current at the free layer of MTJ is given by $h_{ac}{\sim}I_{ac}/2w$, where *w* is the width of the CPW. The current flowing below the MTJ at time *t* depends on the value of m_x at time $t\text{-}\Delta t$, where Δt is the feedback delay. Thus the feedback ac magnetic field acting on the MTJ can be written as:

$$h_{fb}(t) = \frac{\Delta R}{4w(R_T + R_{MTJ})} I_{dc}\, m_x(t - \Delta t) \qquad (4)$$

To obtain the magnetization dynamics, we solve the LLG equation modified to include the effect of feedback magnetic field:

$$\frac{d\hat{m}}{dt} = -\gamma[\hat{m} \times (\overline{H}_{eff} + \overline{H}_{fb} + \overline{h}_r)] - \alpha\gamma[\hat{m} \times \hat{m} \times (\overline{H}_{eff} + \overline{H}_{fb} + \overline{h}_r)] \qquad (5)$$

where *m* demotes unit vector along magnetization, α is the damping constant, γ is related to the gyromagnetic factor γ_0 as $\gamma = \gamma_0/(1+\alpha^2)$. h_r denotes the random magnetic field due to thermal fluctuations with statistical properties:

$$\langle h_{r,i}(t) \rangle = 0, \ \langle h_{r,i}(t) h_{r,j}(s) \rangle = 2D\delta_{ij}\delta(t-s) \qquad (6)$$

$$D = \frac{\alpha k_B T}{\gamma \mu_0 M_s V} \qquad (7)$$

The effective field is given by the sum of external field, exchange field, anisotropy field, demagnetization field. The spin torque term has been neglected in our simulation, as the current applied was small and the feedback magnetic field was amplified by an external amplifier. We have assumed that an amplifier with gain of 21 dB in the feedback path in all simulations. The value of ΔR, R_T and R_{MTJ} were taken as 40 Ω, 50 Ω and 100 Ω respectively. (We also carried out simulations with assuming that the conductance rather than resistance depends on the cosine of the angle between free and fixed layer. The results were similar.) To apply the feedback in the simulation, the average values of m_x were stored after every 10 ps and used later to find out the feedback magnetic field as given by equation S1.

We adjusted the values of anisotropy used in the simulation so as to get a close match of the FMR frequencies with the experimental data. The results are shown in Fig. S1.

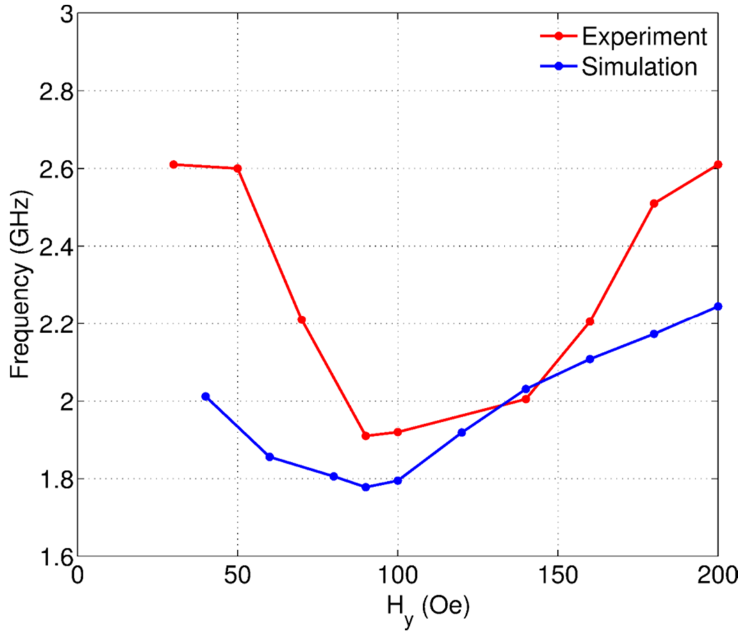

Fig. S1. Frequency of the peak in the noise spectrum as a function of magnetic field applied along y direction. The red curve shows experimental data and blue curve shows micromagnetic simulation results. The noise spectra were measured and simulated by disconnecting feedback waveguide.

The simulation results of spectral density of m_x with feedback delay of 10 and 20 ns is shown in the Fig. S2. The results show presence of side bands. The distance between the side bands is found to be 0.1 GHz and 0.05 GHz respectively for delay of 10 and 20 ns. Thus the sideband separation is given by inverse of the feedback delay time.

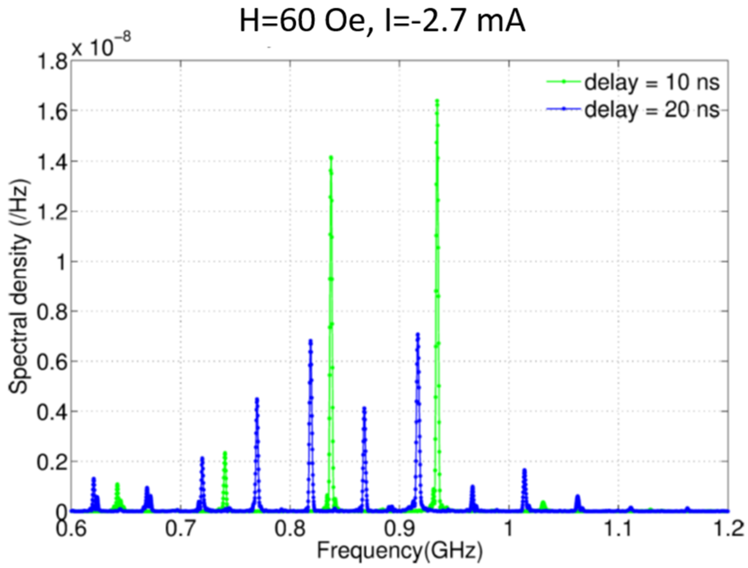

Fig. S2. Simulation results for spectral density for two different feedback delays of 10 and 20 ns. The spectra shows multiple peaks separated by inverse of delay time. An amplifier with gain of 21 dB was assumed to be present in the feedback circuit.

Figure S3 shows the simulation results of the total power as a function of dc current (H=60 Oe). The total power is obtained as:

$$P = [\frac{R_T}{(R_T + R_{MTJ})^2}](0.5 \Delta R I_{dc})^2 \operatorname{var}(m_x) \qquad (8)$$

Where var denotes the variance of average value of m_x, R_T is the termination resistance taken as 50 Ω. One can see that there is a threshold for emission, above which power starts to increase rapidly. At this value of current, we also see a large change in the cross-correlation function shown in Fig. 5b. Thus above this value the different parts of the sample oscillate in-phase and we also get large power output.

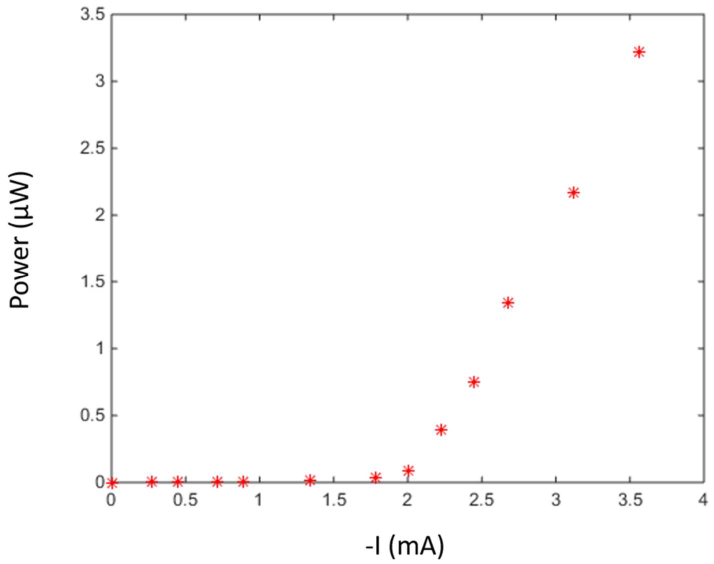

Fig. S3. Simulation Results: Total power given by the device as a function of dc bias current. An amplifier with gain of 21 dB was assumed to be present in the feedback circuit. The device has a threshold current above which the emitted power increases sharply with current.

Supplementary Note 2: Estimation of critical voltage for STT and feedback excitation

We now estimate the critical voltage due to spin transfer torque within single domain approximation (without any feedback.). The LLGS equation is given by:

$$\frac{d\hat{m}}{dt} = -\gamma(\hat{m}\times\overline{H}_{eff}) + \alpha(\hat{m}\times\frac{d\hat{m}}{dt}) + A_{//}(\hat{m}\times\hat{m}\times\hat{M}) \quad (9)$$

$$\overline{H}_{eff} = (H_c m_x + H_{ext})\hat{x} - H_d m_z \hat{z} \quad (10)$$

where γ is the gyromagnetic ratio, α denotes Gilbert damping, $\hat{M}$ denotes unit vector along pinned layer magnetization , $\hat{m}$ denotes unit vector along free layer magnetization. The last term denotes the Slonczewski spin transfer torque term. We assume x to be the easy axis and z as out-of-plane hard axis as indicated by the 2nd equation for effective magnetic field H_{eff}. H_c and H_d denote in-plane and out-of-plane anisotropy fields respectively. The conductance G of the MTJ and the coefficient of the STT term, $A_{//}$ are given by [1]

$$G = G_0(1+P^2 m_x) \quad (11)$$

$$A_{//} = \frac{G_0 P}{q}\frac{\mu_B}{M_s\, volume}\times voltage \quad (12)$$

Where P denotes the polarization, M_s denotes saturation magnetization, q denotes electron charge and μ_B denotes Bohr magneton. The values of G_0 and P obtained from the resistances of parallel and anti-parallel states are $10^{-2}/\Omega$ and 0.45 respectively. Volume of the sample is (500 X 300 X 3) nm^3. The critical voltage V_c is given by:

$$V_c = \alpha\gamma(H_{//} + H_{ext} + \frac{H_\perp}{2})\times\frac{q\, M_s\, volume}{G_0 P \mu_B} \approx \alpha\gamma\frac{H_\perp}{2}\times\frac{q\, M_s\, volume}{G_0 P \mu_B} \quad (13)$$

Taking M_s=800 emu/cm^3, H_d=4πMs, α=0.01 the critical voltage comes out to be about 1.18 V. The maximum voltage used in the present experiment is 0.27 V which is much smaller than V_c.

We now estimate critical current required for feedback oscillations (without any STT) [2]. The critical current, I_c is given by:

$$Ic \approx \frac{\alpha\omega_0}{\gamma f},\ f = \frac{\Delta R}{4w(R_{MTJ}+50)} \tag{14}$$

Where ω_0 is the resonant angular frequency, *w* is the width of feedback line. Using ΔR=40Ω, R_{MTJ}=100 Ω, α=0.01, γ=2.21 X 10^5 m/(A-s), *w*=1 μm, ω_0 =2π x 2.5 GHz, the critical current comes out to be 10.7 mA. As the maximum dc current used in the experiment is 2.7 mA, an amplifier is required in the feedback path.

Supplementary Note 3: Feedback oscillator analysis using universal oscillator model

We here consider a free layer with out of plane uniaxial anisotropy. For the sake of simplicity, the feedback magnetic field is taken as rotating in the x-y plane as described by the equation below:

$$\vec{\mathbf{H}}_{feedback} = -H_{feedback}\left(\sin\theta_f \cos\phi_f \vec{\mathbf{e}}_x + \sin\theta_f \sin\phi_f \vec{\mathbf{e}}_y\right) \tag{15}$$

$$U = U_{ani} + (-\gamma)\vec{\mathbf{S}}\cdot\left(\vec{\mathbf{H}}_{ext} + \vec{\mathbf{H}}_{feedback}\right) \tag{16}$$

$$\vec{\mathbf{H}}_{ext} = -H_{ext}\vec{\mathbf{e}}_z \tag{17}$$

$$-\gamma H_{feedback} = \omega_f,\ \theta_f = \theta(t-\Delta t), \phi_f = \phi(t-\Delta t) \tag{18}$$

The subscript *f* in the above equations refer to feedback and Δt is the feedback delay time. Without the feedback, the system has cylindrical symmetry around z-axis. We now define a dimension-less complex variable c as [3]:

$$c = \frac{1}{\sqrt{2}}\frac{m_x - im_y}{\sqrt{(1+m_z)}} \tag{19}$$

where m_x, m_y and m_z denote components of unit vector along magnetization. The equation of motion for c can be written as:

$$\frac{dc}{dt} \cong (i\Omega_0 - \Gamma_- + \Gamma_+)c + f_f + f_{stochstic} \tag{20}$$

Here,

$$p = |c|^2 \tag{21}$$

$$\Omega_o \equiv \frac{1}{2S_2}\frac{dU_{ani}}{dp},\ \Gamma_- \equiv \alpha(1-p)\Omega_o,\ \Gamma_+ \equiv -\frac{\beta_{ST}}{S_2}(1-p) \tag{22}$$

$$f_f \equiv -i\omega_f\sqrt{1-|c_f|^2}\left(c_f\sqrt{1-|c|^2} - \left(c^* c_f + cc_f^*\right)\frac{c}{2\sqrt{1-|c|^2}}\right) \tag{23}$$

$$f_{stochstic} \equiv -c\left(iF'^1_{stochastic} + \frac{1}{4S_2}\frac{1}{p}F'^2_{stochastic}\right) \tag{24}$$

The first term on RHS in the above equation is the precession term (Ω_0=precession frequency). The second term, involving Γ_- is the dynamic damping term, third term involving Γ_+ is spin-transfer term. The last term denotes random stochastic force, which gives rise to the

broadening of oscillator power spectral density. The fourth term which arises due to the feedback magnetic field is a new term. Without this new term, the above equation describes a "universal auto-oscillator" [1]. We solved the above equation approximately and assuming small feedback delay time and obtained the following expression for the line width:

$$FWHM = \frac{\alpha kT}{4\pi p_0 (1-p_0) S}\left(1+\left(\frac{\Gamma_p}{\Gamma_p + p_0\omega_f}\right)^2 \nu^2 (1-p_0)^2\right)\frac{1}{\left(1+\omega_f \Delta t (1-p_0)\right)^2} \tag{25}$$

In the above equation ν denotes dimensionless frequency shift w.r.t. oscillation amplitude, p_0, α is the Gilbert damping constant and T is temperature. Γ_P is the dynamic damping factor, defined as:

$$\Gamma_p(p_0) = p_0[d(\Gamma_- - \Gamma_+)/dp]_{p_0} \tag{26}$$

We can see from the above equation that the factor of ω_f appears at two places in the denominator of RHS. Thus the feedback effect is very efficient is decreasing the line width. Our analysis shows that feedback reduces thermal noise in power and phase effectively. Dynamic damping is enhanced effectively by a feedback. Therefore, we can have a reduced linewidth. Thus oscillators based on feedback effect can show narrow line widths as experimentally observed in this paper. (The detailed derivation of above expressions will be published later.)

Supplementary Note 4: Experimental Results

The magneto-resistance of the sample measured with in-plane magnetic field applied at 45° is shown in Fig. S4. The device shows a high TMR of 50 %.

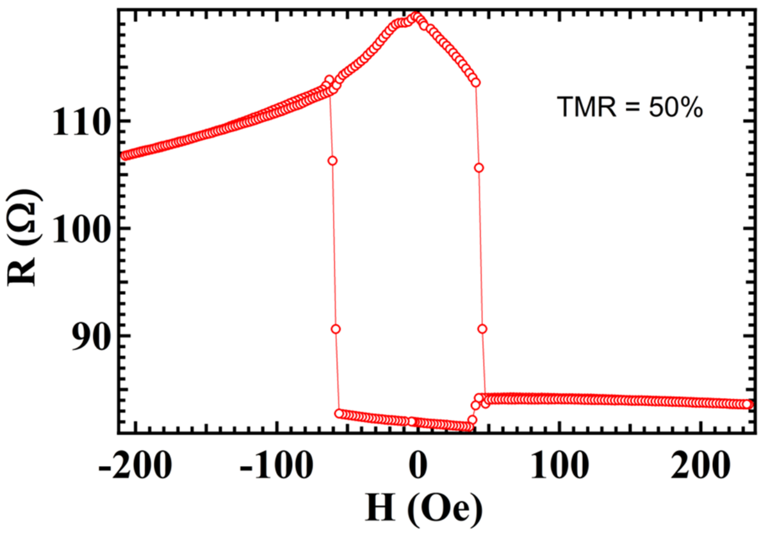

Fig. S4. Experimental data: Tunnel magneto-resistance as a function of in-plane magnetic field applied at 45° to x-axis.

The power spectral density of sample C was measured by inserting an additional cable in the feedback path. The delay of the cable was measured by network analyzer and found to be 8 ns. The power spectra obtained for I = -2 mA, amplifier gain = 27 dB and H = 70 Oe, is shown in Fig. S5 in log scale. The separation between the peaks is now decreased to 62.2 MHz. Without

this extra delay, the peak separation is 120 MHz as shown in Fig. 3a. Thus peak separation is given by the inverse of the round trip delay.

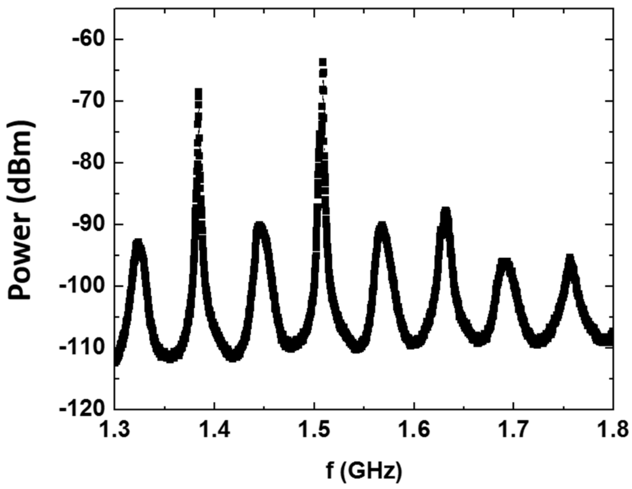

Fig. S5. Experimental data: Power spectra obtained by inserting an additional cable in the feedback path.

Supplementary Note 5: Experimental Results

The power spectra measured on sample A with magnetic field of 58 Oe, for various values of dc currents (from -1.7 mA to -2.7 mA) with feedback amplification of 24 dB on are show in fig 2a. The power spectra measured for the same magnetic field and dc currents but with feedback off are shown in fig S6. Though the values of dc currents here are lower than the estimated threshold current for STT excitation (see supplementary note 2), STT can still affect the power spectra by changing effective damping. Thus the psd amplitude as a function a current shown in fig S6 has two contributions: i) As the ac voltage across MTJ is proportional to dc current, the psd amplitude goes as square of dc current ii) Effects of STT by change in effective damping.

The spectra shown in fig 2a have additional contribution from the feedback effect.The comparison of figures 2a and S6, shows that the feedback gives rise to a large increase in the power output and quality factor.

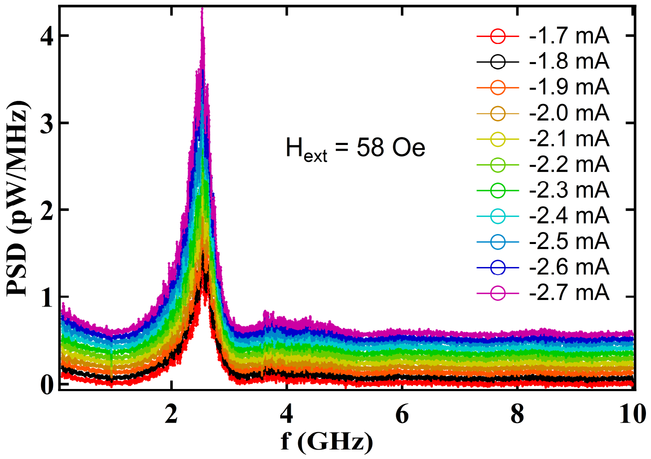

Fig S6. Power spectral density as a function of frequency for various dc currents, without feedback.

Supplementary Note 6: Experimental Results

We inserted a phase shifter (variable delay) in the feedback path (as shown in fig 1a) and measured psd. The measurements were carried out on sample B with magnetic field of 93 Oe, dc current of 1 mA and feedback amplification of 33 dB. The results are shown in the figure S7 for six different values of delays varying from 0 to 134 ps. The spectrum labelled delay 0 corresponds to zero delay setting, spectrum labelled delay 1 corresponds to delay of 22.3 ps etc. When the feedback delay is changed and if the oscillation frequency remains the same, the phase relation between the feedback magnetic field and free layer oscillations changes. The free layer therefore changes its oscillation frequency, so that the optimal phase relationship between feedback magnetic field and free layer oscillations is restored. For large oscillations the frequency depends on amplitude of oscillation due to the non-linear LLG equation. Thus change in frequency is achieved by a change in amplitude. Therefore, both the frequency and amplitude vary with delay. (Experimentally, we also see a sudden jump in the frequency from delay 1 to delay 2.) The maximum adjustable delay used was 134 ps, which corresponds to a phase change of about 116°.

Changing current from positive to negative (see fig 6) corresponds to a phase change of 180°. We can see from figure S7 that the amplitude varies with the delay (i.e. phase), e.g. the ratio of psd amplitude of data labelled with delay1 and delay 2 is about 1.5. The asymmetry of power w.r.t current shown in fig 6, can arise due to the phase change of 180°. There can also be a contribution of STT to the asymmetry.

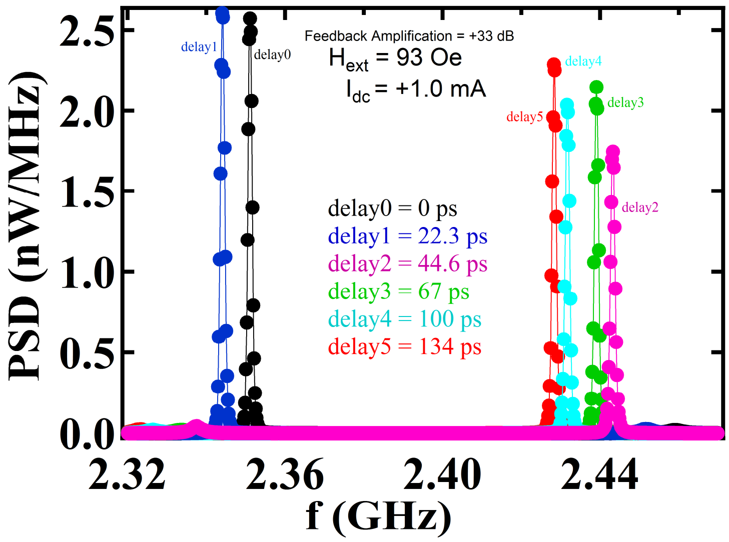

Fig S7. Power spectral density as a function of frequency for various values of delays.

[S1] Slonczewski, J. C., Phys. Rev. B, 39, 6995 (1989)

[S2] Dixit, D. et. al., Appl. Phy. Lett. 101, 122410 (2012).

[S3] Slavin, A. and Tiberkevich, V., IEEE Transactions on Magnetics, 45, 1875 (2009)
